# A Novel L-Phenylalanine Dipeptide Inhibits the Growth and Metastasis of Prostate Cancer Cells via Targeting DUSP1 and TNFSF9

**DOI:** 10.3390/ijms231810916

**Published:** 2022-09-18

**Authors:** Lanlan Li, Mingfei Yang, Jia Yu, Sha Cheng, Mashaal Ahmad, Caihong Wu, Xinwei Wan, Bixue Xu, Yaacov Ben-David, Heng Luo

**Affiliations:** 1 State Key Laboratory for Functions and Applications of Medicinal Plants, Guizhou Medical University, Guiyang 550014, China; 2 The Key Laboratory of Chemistry for Natural Products of Guizhou Province, Chinese Academy of Sciences, Guiyang 550014, China; 3 School of Pharmaceutical Sciences, Guizhou Medical University, Guiyang 550025, China

**Keywords:** L-phenylalanine dipeptide, HXL131, prostate cancer, cell growth and metastasis, proteomics, DUSP1, TNFSF9

## Abstract

Prostate cancer (PCa) is a common malignant cancer of the urinary system. Drug therapy, chemotherapy, and radical prostatectomy are the primary treatment methods, but drug resistance and postoperative recurrence often occur. Therefore, seeking novel anti-tumor compounds with high efficiency and low toxicity from natural products can produce a new tumor treatment method. Matijin-Su [*N*-(*N*-benzoyl-L-phenylalanyl)-*O*-acetyl-L-phenylalanol, MTS] is a phenylalanine dipeptide monomer compound that is isolated from the Chinese ethnic medicine Matijin (*Dichondra repens* Forst.). Its derivatives exhibit various pharmacological activities, especially anti-tumor. Among them, the novel MTS derivative HXL131 has a significant inhibitory effect against prostate tumor growth and metastasis. This study is designed to investigate the effects of HXL131 on the growth and metastasis of human PCa cell lines PC3 and its molecular mechanism through in vitro experiments combined with proteomics, molecular docking, and gene silencing. The in vitro results showed that HXL131 concentration dependently inhibited PC3 cell proliferation, induced apoptosis, arrested cell cycle at the G2/M phase, and inhibited cell migration capacity. A proteomic analysis and a Western blot showed that HXL131 up-regulated the expression of proliferation, apoptosis, cell cycle, and migration-related proteins CYR61, TIMP1, SOD2, IL6, SERPINE2, DUSP1, TNFSF9, OSMR, TNFRSF10D, and TNFRSF12A. Molecular docking, a cellular thermal shift assay (CETSA), and gene silencing showed that HXL131 had a strong binding affinity with DUSP1 and TNFSF9, which are important target genes for inhibiting the growth and metastasis of PC3 cells. This study demonstrates that HXL131 exhibited excellent anti-prostate cancer activity and inhibited the growth and metastasis of prostate cancer cells by regulating the expression of DUSP1 and TNFSF9.

## 1. Introduction

In recent years, the incidence and mortality of cancer have risen and become a significant cause of death concerning human health. The global male cancer statistics report that was released by the International Agency for Research on Cancer (IARC) showed that prostate cancer (PCa) ranked second in male incidence and became the most common form of malignant tumor [1,2,3,4]. Currently, the treatment for PCa patients is mainly drug therapy [5], radical prostatectomy [6], radiotherapy [7,8], chemotherapy [9], and endocrine therapy [10], but often, these face severe challenges such as poor efficacy, complications, and drug resistance. As an essential source of drugs, natural products could be an alternative strategy for preventing and treating cancer diseases. Identifying new anticancer drugs with high efficiency and low toxicity, with a defined function and minimum side-effects from natural product resources could therefore facilitate cancer treatment.

Matijin-Su [*N*-(*N*-benzoyl-L-phenylalanyl)-*O*-acetyl-L-phenylalanol, MTS] is a phenylalanine dipeptide monomer compound with various biological activities, and it was originally isolated from a Chinese ethnic medicine Matijin (*Dichondra repens* Forst.) [11,12]. MTS derivatives display various pharmacological activities, including anti-hepatitis B virus (HBV) [13,14,15], hepatoprotective activities [11,16], anti-inflammatory activities [11], and a promising anti-tumor ability [17,18]. Studies have shown that MTS derivatives 13b, 13c, and 14a showed a significantly higher inhibitory activity on HBV DNA replication than the positive control lamivudine did [19]. Anti-tumor activity evaluation results showed that MTS derivatives 23, 24, and 25 significantly inhibited the proliferation of human hepatocellular carcinoma HepG2 cells, which was superior to the effects of the lead compound MTS. These compounds also exhibited low toxicity to human normal hepatocyte LO2, indicating that MTS derivatives are highly efficient with having a low toxicity [20]. In addition, MTS derivatives 1 and 3 blocked the G1/S phase of the cell cycle by increasing the intracellular reactive oxygen species levels, up-regulating p38 MAPK activity, stabilizing p53, and activating p21 transcription to play a role in anti-hepatoma activity, suggesting that these two MTS derivatives can be used as potential novel hepatocellular carcinoma inhibitors [17].

Based on these results, we have recently designed and synthesized a series of MTS derivatives and systematically evaluated their anticancer activity. This study demonstrates the effects of HXL131, a novel MTS derivative, on the growth and metastasis of PC3 from four aspects: cell proliferation, apoptosis, cell cycle, and migration. The results showed that HXL131 significantly inhibited the growth and metastasis of the PCa cell line PC3. Furthermore, we explored the mechanism of this inhibition of HXL131, and report that DUSP1 and TNFSF9 are therapeutic targets of HXL131, which provides the basis and possibility for targeted cancer therapy.

## 2. Results

### 2.1. Evaluation of the Toxic Effects of HXL131 on Human Regular Hepatic Cell Line LO2

**Figure 1:**
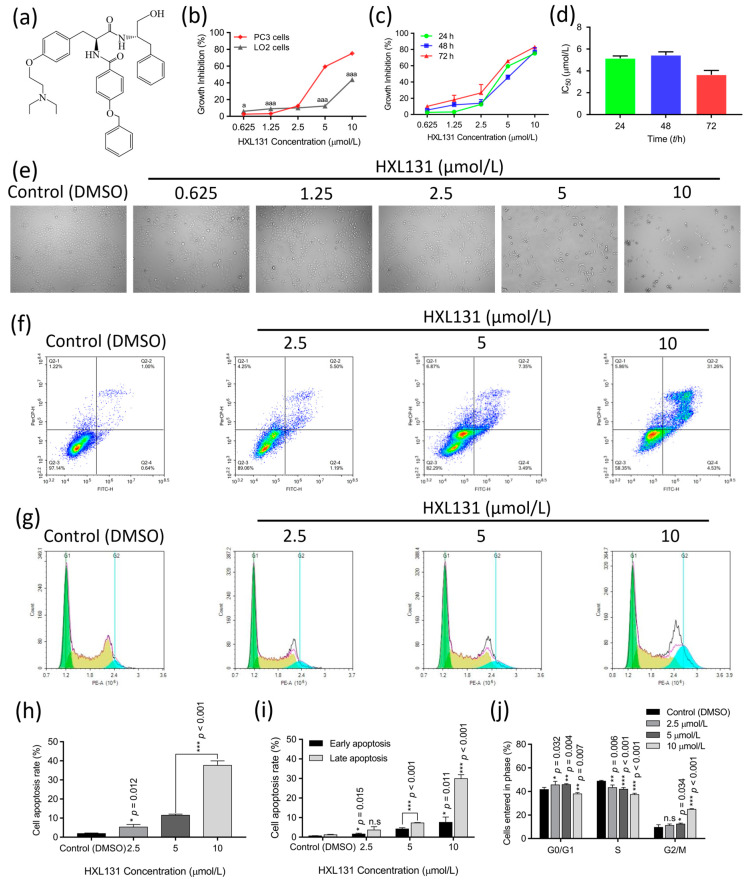
Evaluation of HXL131 toxicity in normal hepatocyte LO2 and the effect of this compound on PC3 cell growth at different concentrations. (**a**) The structural formula of the compound HXL131; (**b**) By comparing the proliferation inhibition of PC3 cells and LO2 cells, HXL131 was more inhibitory in PC3 cells, while indicating less toxicity in LO2 cells; (**c**) A [3-(4,5-dimethylthiazol-2-yl)-2,5-diphenyltetrazolium bromide] (MTT) assay was used to detect the inhibitory effect of different concentrations of HXL131 on the proliferation of PC3 cells; (**d**) The semi-inhibitory concentration (IC_50_) values of HXL131 at different times (24, 48, and 72 h) were calculated by IBM SPSS Statistics 21.0 software; (**e**) Morphological changes of the PC3 cells that were treated with different concentrations of HXL131 for 24 h were observed using an inverted microscope (the magnification = ×100); (**f**) Apoptosis of PC3 cells at different concentrations of HXL131 was determined by flow cytometry; (**g**) Cell cycle changes of PC3 in the control (DMSO) group and HXL131 groups at different concentrations were detected by flow cytometry; (**h**) Total apoptosis rate of PC3 cells that were treated with different concentrations of HXL131; (**i**) Comparison of the effects of different concentrations of HXL131 on the early and late apoptosis rates of PC3 cells; (**j**) After the HXL131 treatment, the percentage of PC3 cells in three different phases of cell cycle was changed. Values are mean ± SD (*n* = 3). * *p* < 0.05, ** *p* < 0.01, *** *p* < 0.001 vs. Control (DMSO) group; ^a^ *p* < 0.05, ^aaa^ *p* < 0.001 vs. PC3 cells group; n.s, non-significant. Multiple *t*-tests and one-way ANOVA were used for statistical analysis.

The compound HXL131 (Figure 1a) is an MTS derivative that was recently isolated in our laboratories. We evaluated the toxicity of HXL131 to LO2 by employing an [3-(4,5-dimethylthiazol-2-yl)-2,5-diphenyltetrazolium bromide] (MTT) assay. The results (Figure 1b) showed that in a 24 h treatment, the inhibitory effect of HXL131 on PC3 cells was significantly higher than that of LO2 cells at both concentrations of 5 and 10 µmol/L. The semi-inhibitory concentration (IC_50_) value of HXL131 was 5.15 ± 0.22 µmol/L for the PC3 cells and 18.59 ± 1.54 µmol/L for the LO2 cells. The selectivity index (SI, IC_50_ of normal cell/IC_50_ of tumor cell) value was 3.61, which was greater than 1 [21], indicating that HXL131 showed good tumor suppressor activity and less hepatotoxicity.

### 2.2. Effect of HXL131 on the Growth of PC3 Cells

#### 2.2.1. Effect of Different Concentrations of HXL131 on the Proliferation of PC3 Cells

An MTT assay showed that the growth inhibition rate of the PC3 cells increased with the increase of the HXL131 concentration in a dose-dependent manner (Figure 1c), and the inhibition rate reached more than 70% at 10 µmol/L. Figure 1d quantitatively analyzed the IC_50_ values at 24 h (5.15 ± 0.22 µmol/L), 48 h (5.43 ± 0.32 µmol/L), and 72 h (3.65 ± 0.39 µmol/L), indicating that the inhibitory effect of HXL131 on the PC3 cells was not time-dependent. Therefore, 24 h was selected as the duration for HXL131 to treat the PC3 cells for subsequent experiments. In a dose-dependent manner, this compound inhibited the cell proliferation and induced the cell fragmentation of PC3 cells in a culture at 24 h (Figure 1e). This result suggests that HXL131 may inhibit the proliferation and induce the apoptosis of PC3 cells.

#### 2.2.2. Effect of Different Concentrations of HXL131 on Apoptosis of PC3 Cells

A flow cytometry analysis showed that there was an increase in the HXL131 concentration and the number of apoptotic cells increased also (Figure 1f). A quantitative analysis in Figure 1h showed that the apoptosis rate of the PC3 cells increased with the increase of the HXL131 concentration in a concentration-dependent manner. Figure 1i showed that the early and late apoptosis rates of the PC3 cells in each concentration group differed significantly when they were compared with those of the control (DMSO). The late apoptosis rate reached 29.92 ± 2.05% at 10 µmol/L, indicating that the HXL131-induced apoptosis in the PC3 cells occurred at the early and late stages, but mainly during late apoptosis. This indicated that HXL131 could inhibit the proliferation of PC3 cells by inducing early and late apoptosis.

#### 2.2.3. Effect of Different Concentrations of HXL131 on the Cycle of PC3 Cells

As shown in Figure 1g, with the increase in HXL131 concentration, the number of cells in the S phase significantly decreased, and the number of cells in the G2/M phase increased. The quantitative analysis results show that (Figure 1j) when they are compared with those of the control (DMSO), the number of cells in the G2/M phase increased with the increase of the HXL131 concentration, indicating that HXL131 interfered with the regulation of cell cycle progression factors and arrested the cell cycle in G2/M phase. The number of S phase cells decreased with the increase of the HXL131 concentration, suggesting that the anti-tumor compound HXL131 inhibited the DNA replication of the PC3 cells. It was concluded that HXL131 inhibited the malignant proliferation of PC3 cells by regulating the S and G2/M phases of the cell cycle.

### 2.3. Effect of HXL131 on PC3 Cell Metastasis

#### 2.3.1. Effect of Different Concentrations of HXL131 on the Wound-Healing Ability of PC3 Cells

**Figure 2:**
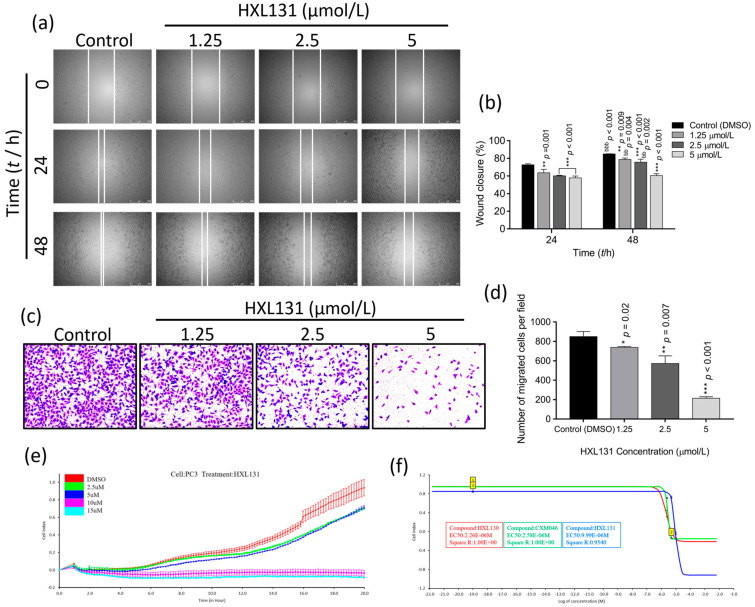
Effects of different concentrations of HXL131 on PC3 cell migration and safety evaluation of three compounds. (**a**) The effects of different times (0, 24, and 48 h) and concentrations of HXL131 (0, 1.25, 2.5, and 5 µmol/L) on wound-healing of PC3 cells were observed under an inverted microscope (the magnification = ×50, scale bar represents 500 µm); (**b**) Statistical results showed that HXL131 inhibited PC3 cell migration in a time- and concentration-dependent manner; (**c**) The effect of HXL131 on the ability of PC3 cells to penetrate the basement membrane of the trans-well chamber was observed using an inverted phase contrast microscope (the magnification = ×100); (**d**) Statistics of changes in the number of PC3 cells passing through the basement membrane of the trans-well chamber after the HXL131 treatment; (**e**) A real-time cellular analysis (RTCA) monitored the changing trend of HXL131’s effect on PC3 cell migration in real-time; (**f**) RTCA software analyzed the median effective concentration (EC_50_) values of the PC3 cells that were treated with the three compounds for 20 h. Values are mean ± SD (*n* = 3). * *p* < 0.05, ** *p* < 0.01, *** *p* < 0.001 vs. Control (DMSO) group; ^bb^ *p* < 0.01, ^bbb^ *p* < 0.001 vs. 24 h group; Multiple *t*-tests and two-way ANOVA were used for statistical analysis.

Figure 2a illustrates the migration of the PC3 cells at different treatment intervals and concentrations, respectively. As shown in Figure 2b, HXL131 inhibited the migration of the PC3 cells in a concentration-dependent manner at both 24 h and 48 h. In addition, the degree of mobility at 48 h was significantly different at the same concentration to that at 24 h, suggesting that the inhibition of cell migration by HXL131 is also time-dependent. Collectively, HXL13 inhibits PC3 cell migration in a concentration- and time-dependent manner.

#### 2.3.2. Effect of Different Concentrations of HXL131 on the Migration of PC3 Cells

Changes in the number of cells that were stained by crystal violet were observed under an inverted phase-contrast microscope, and the results are shown in Figure 2c. The number of stained cells across the basement membrane of the trans-well chamber decreased gradually with an increasing HXL131 concentration. The quantitative analysis results showed (Figure 2d) that when they were compared with those of the control (DMSO), the number of cells that were crossing the membrane was significantly reduced at 1.25, 2.5, and 5 µmol/L concentrations, with significant differences. The analysis showed that HXL131 could significantly migrate PC3 cells in a concentration-dependent manner.

#### 2.3.3. Effect of Different Concentrations of HXL131 on Real-Time Migration of PC3 Cells

The abovementioned results have also confirmed that HXL131 has a significant indigenous inhibitory effect on the proliferation of PC3 cells. Real-time cellular analysis (RTCA) technology was used to monitor the migration process of the PC3 cells in real-time to analyze further the effect of this compound on the metastasis of cancer cells. As shown in Figure 2e, when they were compared with those of the control (DMSO), the number of migrating cells increased with the extension of the monitoring time at 2.5 µmol/L and 5 µmol/L concentrations, thus presenting a synchronous growth trend. However, real-time cell migration was not detected at 10 µmol/L and 15 µmol/L concentrations, indicating that HXL131 inhibited the real-time migration process of PC3 cells in a concentration-dependent manner.

In addition, the median effective concentration (EC_50_) determination of three MTS derivatives with good activity was also performed in this study to evaluate their safety. RTCA Software was used to calculate the EC_50_ values of the three compounds against PC3 cells at 20 h, and the results were 2.26 µmol/L (HXL130, R^2^ = 1.00), 2.58 µmol/L (CXM046, R^2^ = 1.00) and 9.99 µmol/L (HXL131, R^2^ = 0.9540), respectively, thus showing an excellent linear relationship. The analysis showed (Figure 2f) that among the three MTS derivatives with significant tumor-suppressive effect, HXL131 had the highest safety, indicating its high efficiency, low toxicity, and value for further study.

### 2.4. Effect of HXL131 on Protein Expression of PC3 Cells Was Analyzed by TMT Quantitative Proteomics

To further analyze the molecular mechanism that HXL131 use to inhibit the growth and metastasis of PC3 cells, this study used Tandem mass tag (TMT) labeling-based quantitative proteomics sequencing technology to search for differentially expressed proteins (DEPs) between HXL131 and the control (DMSO) group to reveal its potential molecular mechanism.

#### 2.4.1. Expression of Differential Proteins in the HXL131 Treatment Group

**Figure 3:**
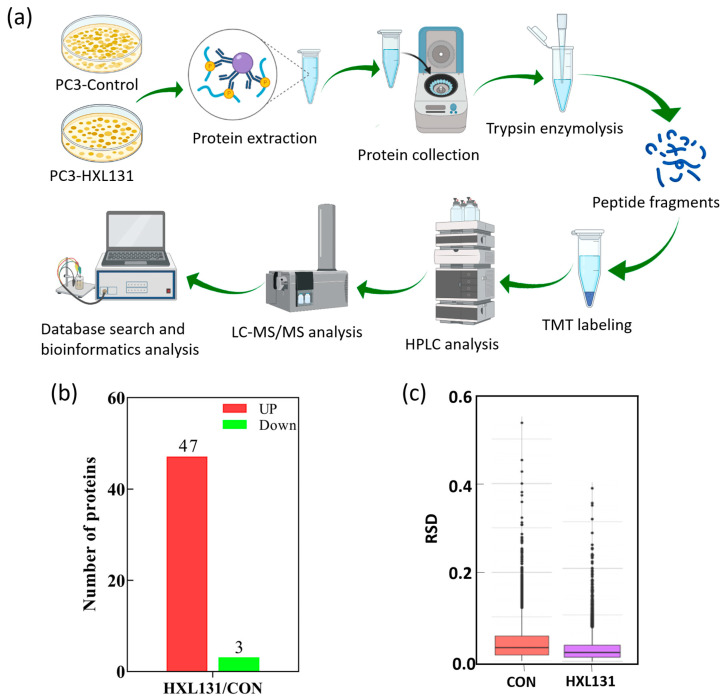
Tandem mass tag (TMT) quantitative proteomics analysis of differentially expressed proteins (DEPs). (**a**) Systematic workflow diagram of TMT labeling-based quantitative proteomic analysis. HPLC: High-performance liquid chromatography. LC-MS/MS: Liquid chromatography-tandem mass spectrometry; (**b**) The number of up-regulated and down-regulated expression proteins (Fold change > 2.0); (**c**) Quantitative relative standard deviation (RSD) of control samples and HXL131-treated samples.

The flow chart of the TMT labeling-based quantitative proteomics procedure is shown in Figure 3a. According to the sequencing results, when they were compared with those of the control (DMSO), 2196 DEPs were identified in the HXL131 group, of which 1111 DEPs were up-regulated, and 1085 were down-regulated. Under a fold change > 2.0 and a *p*-value < 0.05, 50 DEPs were obtained (Table 1), including 47 up-regulated proteins and three down-regulated proteins (Figure 3b). We used relative standard deviation (RSD) to assess the repeatability of the protein quantification. Figure 3c is a boxplot of the RSD for the quantitative protein values of the control and HXL131 samples. When the overall RSD value is smaller, the quantitative repeatability is better, and therefore, the results are more reliable.

#### 2.4.2. Functional Classification of DEPs

**Figure 4:**
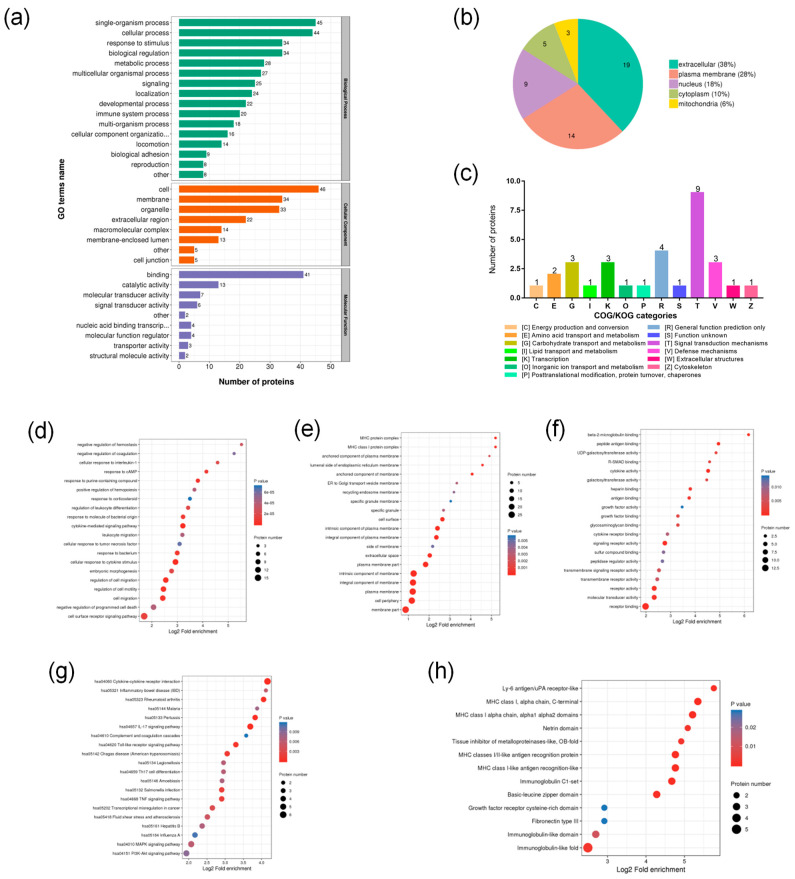
Functional classification and enrichment analysis of DEPs. (**a**) Statistical distribution of DEPs under each Gene Ontology (GO) category, including biological process (BP), cellular component (CC), and molecular function (MF); (**b**) Subcellular localization of DEPs; (**c**) Clusters of Orthologous Groups of proteins/euKaryotic Ortholog Groups (COG/KOG) functional classification of DEPs; (**d**) BP in GO enrichment; (**e**) CC in GO enrichment; (**f**) MF in GO enrichment; (**g**) Kyoto Encyclopedia of Genes and Genomes (KEGG) pathway enrichment analysis of DEPs; (**h**) Protein domain enrichment analysis of DEPs.

The identified DEPs were classified using Gene Ontology (GO) secondary annotations, subcellular structure localization, and the Clusters of Orthologous Groups of proteins/euKaryotic Ortholog Groups (COG/KOG) function to explore their distribution in different functions. GO explains the role of the DEPs from the perspectives of a biological process (BP), a cellular component (CC), and a molecular function (MF). Figure 4a show that in the GO secondary annotation procedure, the DEPs in the HXL131 treatment group mainly included the single-organism process, a response to stimulus, biological regulation, and the metabolic process in the BP category. Other processes such as multi-organism processes, locomotion, biological adhesion, and reproduction were also affected by HXL131. In the CC category, the most enriched proteins were related to the cell, membrane, organelle, and extracellular region. In the MF category, the differential proteins were mainly concentrated in the processes of binding, catalytic activity, molecular, and signal transducer activity. Subcellular structure localization was used to find the specific location of the DEPs in the cells. Figure 4b shows that the DEPs were mainly distributed in the extracellular region (38%), plasma membrane (28%), nucleus (18%), cytoplasm (10%), and mitochondria (6%), indicating that the DEPs after the HXL131 treatment were mainly extracellular proteins and membrane proteins. Generally, the COG is divided into two categories: one is prokaryotic, and the other is eukaryotic. Prokaryotes are called the COG database, and eukaryotes are called the KOG database. The COG/KOG functional classification (Figure 4c) showed that DEPs were mainly enriched in signal transduction mechanisms, general function prediction only, carbohydrate transport and metabolism, and transcription and defense mechanisms.

#### 2.4.3. Functional Enrichment Analysis of DEPs

The enrichment analysis was conducted on the function of the DEPs from the GO classification, Kyoto Encyclopedia of Genes and Genomes (KEGG) pathway, and protein domain to discern whether DEPs have a significant enrichment trend in some functional types. In the BP of the GO enrichment classification, the DEPs were mainly enriched in the cell surface receptor signaling pathways, the negative regulation of programmed cell death, cell migration, the regulation of cell motility, the cellular response to cytokine stimulus, and the cellular response to cytokine stimulus (Figure 4d). The CC of the DEPs were enriched in the membrane, cell periphery, plasma membrane, and the integral and intrinsic components of the membrane (Figure 4e). The MF of the DEPs were mainly enriched in the receptor binding activity, molecular transducer activity, transmembrane receptor activity, signaling receptor activity, and cytokine activity (Figure 4f). In the KEGG pathway (Figure 4g), the DEPs were involved in regulating 20 signaling pathways which were mainly enriched in PI3K-Akt, MAPK, TNF, Toll-like receptor, IL-17, and cytokine–cytokine receptor interaction signaling pathways. Protein domain enrichment (Figure 4h) showed that the DEPs were mainly enriched in the immunoglobulin-like fold, Basic leucine zipper domain, Immunoglobulin C1-set, MHC class I-like antigen recognition-like, MHC classes I/II-like antigen recognition protein, MHC class I alpha chain, alpha1 alpha2 domains, MHC class I, alpha chain, C-terminal, and the Ly-6 antigen/uPA receptor-like domain.

#### 2.4.4. PPI Network Analysis of DEPs

**Figure 5:**
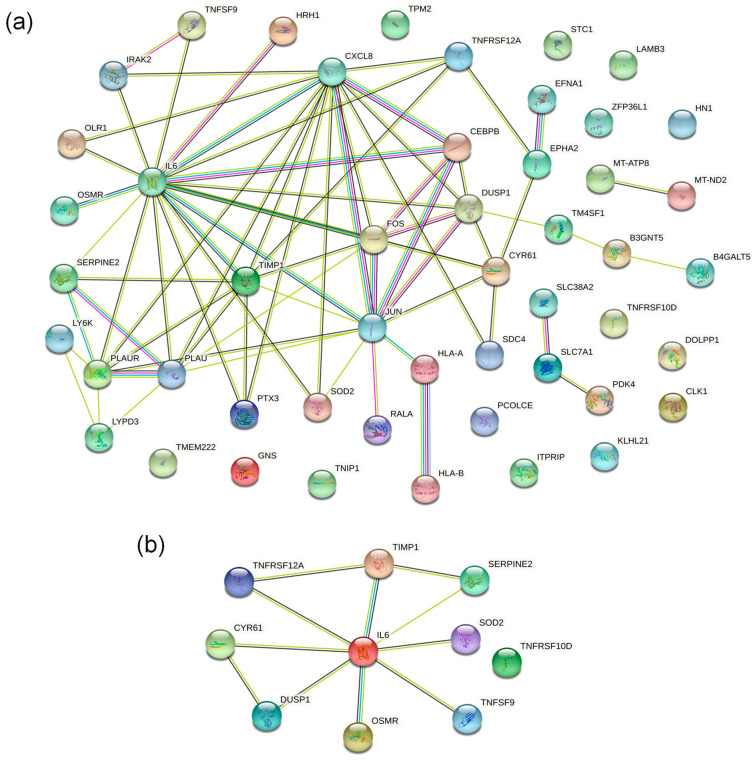
Protein–protein interaction (PPI) network analysis of DEPs. (**a**) PPI network results of 50 DEPs. IL6, TIMP1, CXCL8, DUSP1, and other DEPs played a central role; (**b**) PPI network results of 10 key DEPs. IL6 plays a significant regulatory role in the 10 key DEPs.

Under a fold change > 2.0, the interaction relationship between 50 DEPs was drawn and visualized using the STRING (v.11.5) protein–protein interaction (PPI) network database. The visualization results (Figure 5a) showed that IL6, TIMP1, CXCL8, PLAU, JUN, FOS, CYR61, DUSP1, and other DEPs played a central role in the PPI network.

### 2.5. Screening of Essential Proteins Regulated by HXL131

Based on the abovementioned GO enrichment analysis, KEGG enrichment analysis, protein domain enrichment analysis, and PPI network analysis, we screened 10 key DEPs that regulate the proliferation, apoptosis, cycle, and migration of PC3 cells. They were CYR61, TIMP1, SOD2, IL6, SERPINE2, DUSP1, TNFSF9, OSMR, TNFRSF10D, and TNFRSF12A (Figure 5b). Their expression was significantly increased in the HXL131-treated PC3 cells. The results of the PPI network showed that IL6 plays a significant regulatory role in 10 key DEPs.

### 2.6. Validation of 10 Key DEPs

#### 2.6.1. The GEPIA Database Verifies the Expression of 10 Key DEPs

**Figure 6:**
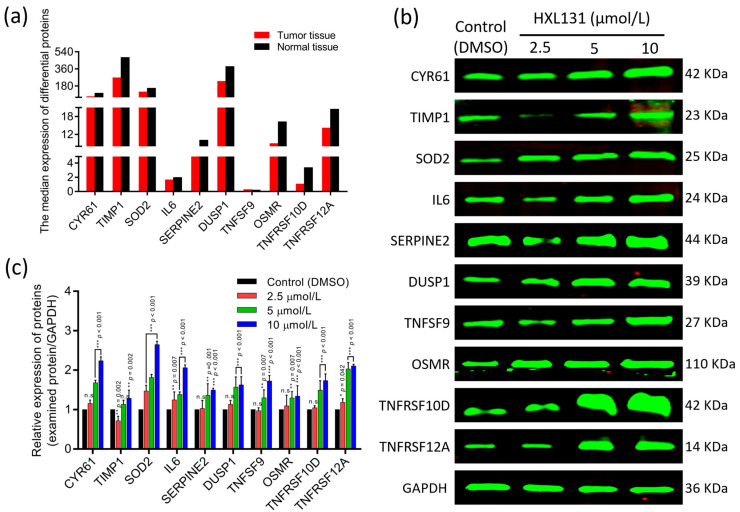
A Gene Expression Profiling Interactive Analysis (GEPIA) database and a Western blot validate the expression of the 10 key DEPs. (**a**) The expression of 10 key DEPs in prostate cancer (PCa) tissues and adjacent normal tissues was verified by the GEPIA database; (**b**) A Western blot band map developed using the Odyssey infrared imaging system; (**c**) Statistical plots of the relative expression of proteins. Values are mean ± SD (*n* = 3). * *p* < 0.05, ** *p* < 0.01, *** *p* < 0.001 vs. Control (DMSO) group; n.s, non-significant. Multiple *t*-tests and two-way ANOVA were used for statistical analysis.

The expression of these 10 critical DEPs in PCa tissues and adjacent normal tissues was verified by a Gene Expression Profiling Interactive Analysis (GEPIA). The data analysis showed that (Figure 6a), except for the expression of TNFSF9 in PCa tissues and adjacent normal tissues which was similar, the expression of other proteins in the adjacent normal tissues was higher than that in the PCa tissues, indicating that these factors are inhibitory factors for the occurrence and development of PCa. The up-regulation of its expression can significantly inhibit the malignant development and metastasis of PCa.

#### 2.6.2. Western Blot Verified the Expression of 10 Key DEPs

We further verified the proteomics and GEPIA database results at the protein level using an immunoblotting technique. The results in Figure 6b and Figure 6c show that when they were compared with those of the control (DMSO), the relative expression levels of 10 essential proteins in the HXL131 treatment group were significantly different, and the protein expression levels increased with the increase of the concentration in a concentration-dependent manner. The analysis showed that the protein expressions of CYR61, TIMP1, SOD2, IL6, SERPINE2, DUSP1, TNFSF9, OSMR, TNFRSF10D, and TNFRSF12A were increased in the HXL131 treatment group, which verified the results of the proteomics and GEPIA database.

#### 2.6.3. Molecular Docking Verified the Targeted Binding between the Target Protein and Small-Molecule Compound HXL131

**Figure 7:**
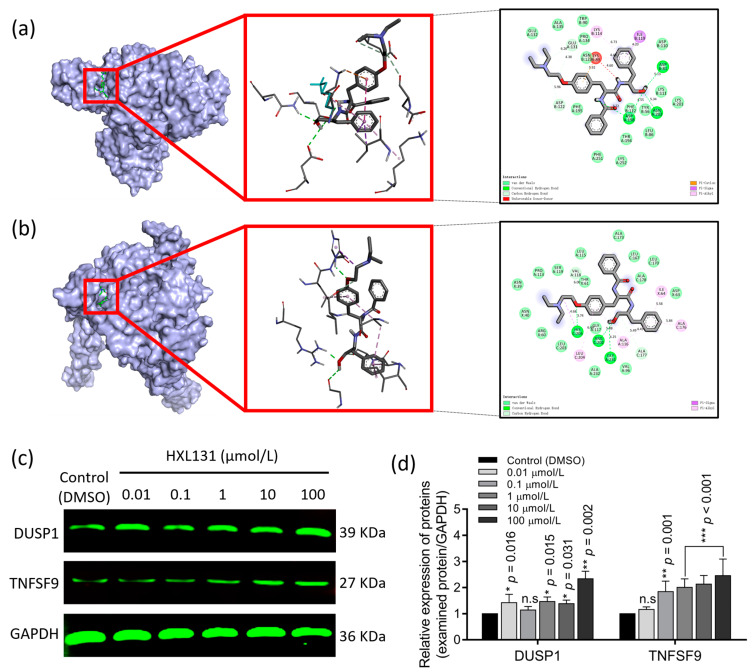
Validation of the two targets of HXL131 regulation, DUSP1, and TNFSF9. (**a**) Molecular docking visualization model of small-molecule ligand (compound HXL131) with the protein receptor (DUSP1). This compound formed hydrogen bonds with ASP A: 198, ASP B: 81, and ASN A: 202 amino acid residues site; (**b**) Molecular docking visualization model of the small-molecule ligand (compound HXL131) with the protein receptor (TNFSF9). The compound formed hydrogen bonds with HIS C: 205, ARG C: 202, and GLY A: 231 amino acid residue site; (**c**) The Odyssey infrared imaging system shows a band map; (**d**) Statistical plots of the relative expression of DUSP1 and TNFSF9 proteins. The targeted binding ability of HXL131 to target the DUSP1 and TNFSF9 proteins was verified by (**c**,**d**) when we were using a cellular thermal shift assay (CETSA). Values are mean ± SD (*n* = 3). * *p* < 0.05, ** *p* < 0.01, *** *p* < 0.001 vs. Control (DMSO) group; n.s, non-significant. Multiple *t*-tests and two-way ANOVA were used for statistical analysis.

The entry and PDB ID of the 10 key DEPs, and the binding affinity between the small-molecule ligands (HXL131) and the protein receptors (CYR61, TIMP1, SOD2, IL6, SERPINE2, DUSP1, TNFSF9, OSMR, TNFRSF10D, and TNFRSF12A) are listed in Table 2. In the docking results, the lower the binding affinity value was, then the higher the binding energy of the ligand to the target was [22]. Therefore, the two docking results with the lowest binding energy were selected for a visual analysis, and these were, respectively, DUSP1 (Figure 7a) and TNFSF9 (Figure 7b). The visual analysis shows that when the ligand HXL131 and the receptor DUSP1 protein interact, the compound forms hydrogen bonds with three amino acid residues sites, ASP A: 198, ASP B: 81, and ASN A: 202. When the targeted binding of HXL131 to TNFSF9 proteins occurs, the compound forms hydrogen bonds with three amino acid residues sites, HIS C: 205, ARG C: 202, and GLY A: 231.

#### 2.6.4. CETSA Verified the Targeted Binding of HXL131 to DUSP1 and TNFSF9 in Molecular Docking

When a target protein binds to a drug molecule, it usually becomes stable and less susceptible to thermal denaturation. This change can be seen in the cellular thermal shift assay (CETSA) results. Therefore, the DUSP1 and TNFSF9 target proteins with a low binding energy were selected for the CETSA analysis. The results showed that (Figure 7c,d) when they were compared with those of the control (DMSO), the protein expression levels of DUSP1 and TNFSF9 were significantly increased. Still, the expression levels of DUSP1 did not increase with the increase of the compound concentration, while the expression levels of TNFSF9 increased with the increase of the compound concentration. These results suggest that HXL131 could stably bind DUSP1 and TNFSF9 proteins, and the degree of binding to TNFSF9 was concentration-dependent.

#### 2.6.5. Gene Silencing Validated the Effect of Interfering with the Expression of DUSP1 and TNFSF9 on PC3 Cell Growth and Migration

**Figure 8:**
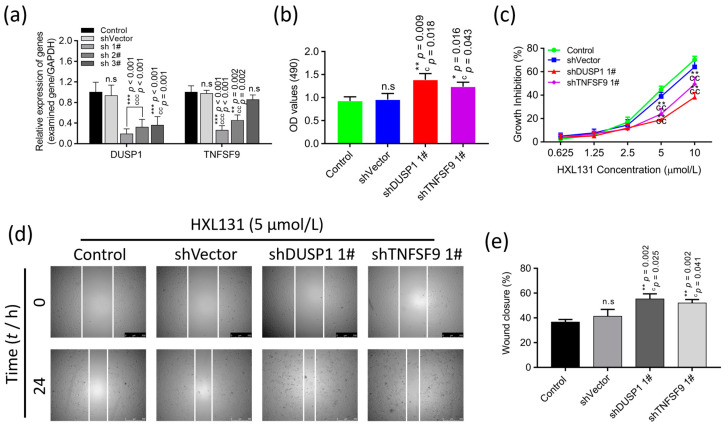
Gene silencing validates the effect of interfering with the expression of DUSP1 and TNFSF9 on the growth and metastasis of PC3 cells. (**a**) Real-time quantitative PCR (RT-qPCR) to verify the interference effect of three sequences; (**b**) OD values of the cells in each group were detected by the MTT method at 24 h. This value is proportional to the number of viable cells; (**c**) MTT assay was used to detect the effect of HXL131 on cell proliferation in each group; (**d**) Wound-healing assay was used to detect the effect of HXL131 on cell migration in each group (the magnification = ×50, scale bar represents 500 µm); (**e**) shDUSP1 1# and shTNFSF9 1# promotes migration of PC3 cells at the scratch. “Control” indicates no transfection of any plasmid, “shVector” indicates the empty vector transfected without the target plasmid, “shDUSP1 1#” indicates that plasmid 1# containing DUSP1 was transfected, and “shTNFSF9 1#” indicates that plasmid 1# containing TNFSF9 was transfected. Values are mean ± SD (*n* = 3). * *p* < 0.05, ** *p* < 0.01, *** *p* < 0.001 vs. Control group; ^c^ *p* < 0.05, ^cc^ *p* < 0.01, ^ccc^ *p* < 0.001 vs. shVector group; n.s, non-significant. Multiple *t*-tests and one-way ANOVA were used for statistical analysis.

We performed gene silencing assays to examine the effect of interfering with DUSP1 and TNFSF9 mRNA expression on the growth and metastasis of PC3 cells. The plasmids containing the DUSP1 and TNFSF9F target genes were transfected into the PC3 cells, and the transfection efficiency was verified by a real-time quantitative PCR (RT-qPCR) 48 h later. The results showed (Figure 8a) that when they were compared with that of the control group and the shVector group, the mRNA expression levels of DUSP1 and TNFSF9 were significantly reduced. Among them, shDUSP1 1# and shTNFSF9 1# have the best interference effect and were used in the subsequent experiments.

Then, the effect of shDUSP1 1# and shTNFSF9 1# on the growth and migration of the PC3 cells were examined by an MTT and a wound-healing assay. The results showed (Figure 8b) that silencing the expression of DUSP1 and TNFSF9 promoted the growth and proliferation of the prostate cancer PC3 cells. After a treatment with compound HXL131, the proliferation inhibition rates of all groups increased, but the inhibition rates of shDUSP1 1# and shTNFSF9 1# groups were significantly lower than those of the control and shVector groups (Figure 8c). These results indicated that DUSP1 and TNFSF9 were the direct targets of HXL131 in inhibiting the proliferation of the prostate cancer cells. The wound-healing assay results showed (Figure 8d,e) that cells migrated in all groups at 24 h when they were compared with those at 0 h, and the migration rates of shDUSP1 1# and shTNFSF9 1# groups were significantly higher than those of the control and shVector groups. These results indicate that HXL131 can inhibit the growth and metastasis of prostate cancer PC3 cells by targeting DUSP1 and TNFSF9, suggesting that DUSP1 and TNFSF9 are the direct targets of HXL131.

## 3. Discussion

In this study, we investigated the molecular mechanism that the MTS derivative HXL131 uses to regulate the growth and metastasis of PCa cell lines PC3. We found that HXL131 significantly inhibited the growth and migration process of the PC3 cells, induced apoptosis, and blocked the G2/M phase of the cell cycle in a concentration-dependent manner. TMT labeling-based quantitative proteomics, GEPIA data, and a Western blot indicated the molecular mechanism of HXL131 against prostate cancer. HXL131 inhibited the growth and metastasis of PC3 cells by upregulating the protein levels of CYR61, TIMP1, SOD2, IL6, SERPINE2, DUSP1, TNFSF9, OSMR, TNFRSF10D, and TNFRSF12A. Molecular docking, CETSA, and a gene silencing assay suggested that DUSP1 and TNFSF9 are the critical targets for HXL131 action. The abovementioned results showed that the novel MTS derivative HXL131 significantly inhibited PC3 cell growth and metastasis, and it has anti-PCa solid activity. The ten targeted genes that were coupled with IL6 as the core can be used for HXL131 therapy, with DUSP1 and TNFSF9 as the critical targets for regulation, thus laying the foundation and possibility for their use in the targeted therapy of PCa.

DUSP1 was first to be identified in cultured mouse cells as a member of the threoninetyrosine dual-specificity phosphatase family [23]. It not only participates in the proliferation, apoptosis, and differentiation of normal cells, but also plays a vital role in tumor cells, such as inhibiting the metastasis and invasion of pancreatic, gallbladder, and other malignant tumor cells and inducing the apoptosis of prostate cancer cells [24,25]. DUSP1 promotes the apoptosis of PCa cells by inhibiting the p38 MAPK/NF-kB signaling pathway [25,26,27,28,29]. In addition, it inhibits cell migration and invasion by down-regulating the Snail-inactivated JNK and ERK pathways, and it serves as a marker for the prognosis of PCa [30]. In this study, the proteomic results showed that DUSP1 was one of the critical DEPs that was regulated by HXL131, and the molecular docking results showed that HXL131 and DUSP1 had a low binding affinity. Therefore, we predict that DUSP1 is a crucial target of HXL131 in regulating cell growth and metastasis. Further, the CETSA and gene silencing results showed that the thermal stability of HXL131 was enhanced after binding to the target protein DUSP1, and the growth and metastasis ability of the PC3 cells was significantly enhanced after silencing the expression of DUSP1. These results indicated that DUSP1 could be a key target against the progression of prostate cancer.

As one of the members of the TNF superfamily, TNFSF9 is lowly expressed in human hepatocellular carcinoma tissues. The overexpression of TNFSF9 and the treatment of the recombinant TNFSF9 protein can significantly inhibit the proliferation, metastasis, and invasion of hepatocellular carcinoma cells Huh7 and SMMC-7721 in vitro, and the tumor-suppressive effect of TNFSF9 was also demonstrated in vivo so that it can be used as a tumor-inhibiting factor of hepatocellular carcinoma [31]. However, another report showed that the expression of TNFSF9 in pancreatic cancer, leukemia and lymphoma, gastric cancer, renal cancer, and colorectal cancer was higher than that in adjacent normal tissues, indicating that TNFSF9 acts as an oncogenic factor in these cancers [32]. Therefore, TNFSF9 may play different roles in different cancers. In this study, TNFSF9 was up-regulated in the HXL131-treated PC3 cells, thus acting as a tumor suppressor. Similarly, we used gene silencing assays to interfere with the TNFSF9 mRNA expression. The results showed that the PC3 cells that were transfected with a TNFSF9 interference plasmid had significantly enhanced growth and metastasis ability when they were compared with the PC3 cells without any interference plasmid transfection, indicating that TNFSF9 could be used as a target molecule for HXL131 to inhibit the growth and metastasis of PC3 cells.

In addition, CYR61, TIMP1, SOD2, IL6, SERPINE2, OSMR, TNFRSF 10D, and TNFRSF12A are also key DEPs that are regulated by HXL131. CYR61, as an essential extracellular matrix regulator, regulates cell growth, differentiation, adhesion, angiogenesis, apoptosis, and migration [33,34,35]. The expression of CYR61 mediates the apoptosis of PCa cells and lung cancer cells, inhibits proliferation, and arrests cells in the G0/G1 phase [36]. The increased apoptosis of endometrial cancer cells that are induced by CYR61 overexpression is related to the increased expression of pro-apoptotic proteins Bax, Bad, and TNF-related apoptosis-inducing ligand (TRAIL) [34]. However, other studies have shown that CYR61 acts as an oncogene in breast cancer and squamous cell carcinoma, thus promoting the collective migration of cancer cells and the formation of invasive tumor nests [35,37]. TIMP1 is a matrix-metalloproteinase inhibitor. As an aging gene, it functions in an opposite manner to that of the MMP family of migration proteins, which can inhibit the invasion and metastasis of tumor cells and promote apoptosis [38]. As an antioxidant factor, a high expression of SOD2 can enhance the ability of tumor cells to clear reactive oxygen species and inhibit malignant tumor growth [39]. The overexpression of SOD2 in breast cancer MCF-7 cells increased the expression of p21 and E-cadherin and decreased the expression of Cyclin D1 and MMP-2, suggesting that SOD2 inhibits the proliferation, migration, and invasion of MCF-7 cells [40,41,42]. IL6 is an inflammatory factor with multiple functions in both immune and non-immune cells. Its abnormal signal transduction is involved in the pathogenesis of autoimmune diseases, inflammation, and various cancers [43,44]. Many kinds of literature show that the occurrence and development of tumors are related to the abnormal activation of IL6 [45,46,47,48]. However, there are also reports that IL6 is an inhibitor of tumor cell growth, which functions to inhibit tumor cell growth by activating the anti-tumor T cell immune response [49]. SERPINE2 is a protein that is secreted into the extracellular matrix, and it inhibits cell invasion in PCa and glioma, which is related to the inhibition of uPA, MMP-2, and MMP-9 activities [50,51]. OSMR may be a tumor suppressor in colorectal cancer, and methylation OSMR may play a catalytic role in non-invasive colorectal cancer [52]. As a tumor suppressor receptor, OSMR can inhibit the growth of tumor cells and induce their differentiation in various ways; this is a cytokine with good application prospects [53]. TNFRSF10D is negatively correlated with the biochemical recurrence risk score of prostate cancer, suggesting that the overexpression of TNFRSF10D can reduce the incidence of biochemical recurrence [54]. As an aging gene, the decreased expression of TNFRSF12A in thyroid cancer indicates a poor prognosis. The PPAR signaling pathway, the insulin signaling pathway, and the mTOR signaling pathway may be the critical pathways for controlling TNFRSF12A in thyroid cancer [55]. TRAIL can induce the apoptosis of multiple primary tumor cells such as osteosarcoma without affecting the adjacent normal cells, which has become an effective strategy for tumor treatment [56,57].

In summary, the MTS derivative HXL131 has significant anti-PCa activity. Target molecules CYR61, TIMP1, SOD2, IL6, SERPINE2, DUSP1, TNFSF9, OSMR, TNFRSF10D, and TNFRSF12A play vital roles in regulating cell proliferation, apoptosis, cycle and migration, and they can be used as biomarkers for the treatment of PCa by the MTS derivative HXL131. DUSP1 and TNFSF9 serve as key regulatory targets of HXL131, this providing new candidate targets for the clinical treatment of PCa.

## 4. Materials and Methods

### 4.1. Chemicals, Reagents, and Instruments

Dulbecco’s Modified Eagle Medium (DMEM) was purchased from Gibco (Waltham, MA, USA). Trypsin was purchased from Biological Industries (Kibbutz Beit-Haemek, Israel). MTT was purchased from Solarbio (Beijing, China). Dimethyl sulfoxide (DMSO) was purchased from Zhiyuan Chemical Reagent Co., Ltd. (Tianjin, China). Cell apoptosis and cycle kits were purchased from BD Biosciences (San Jose, CA, USA). The trans-well chambers were purchased from Corning (Corning, NY, USA). The BCA protein assay kit was purchased from beyotime (Shanghai, China). Other reagents and solvents were of analytical grade or commercially available and used without further purification. The CO_2_ cell incubator was purchased from Thermo Fisher Scientific (Waltham, MA, USA). The allegraX-15R high-speed centrifuge was purchased from Beckman (Brea, CA, USA). The microplate reader was purchased from BioTek (Winooski, VE, USA). ACEA NovoCyte flow cytometry analyzer was purchased from BD Biosciences (USA). The Leica inverted fluorescence microscope was purchased from Leica (Wetzlar, Germany).

### 4.2. Antibodies

All antibodies were purchased from Abcam (Cambridge, UK), and they were used as follows: CYR61 (no. ab230947, diluted: 1:1000), TIMP1 (no. ab211926, diluted: 1:1000), SOD2 (no. ab68155, diluted: 1:1000), IL6 (no. ab233706, diluted: 1:1000), SERPINE2 (no. ab134905, diluted: 1:1000), DUSP1 (no. ab61201, diluted: 1:1000), TNFSF9 (no. ab68185, diluted: 1:1000), OSMR (no. ab282577, diluted: 1:1000), TNFRSF10D (no. ab108421, diluted: 1:1000), TNFRSF12A (no. ab109365, diluted: 1:1000) and GAPDH (no. ab181602, diluted: 1:1000). The anti-rabbit IgG (H + L) (Dylight (TM) 800 4 × PEG Conjugate) secondary antibodies that were used in this study were purchased from Cell Signaling Technology and used at a 1:30,000 dilution in the experiments.

### 4.3. Cell Culture and Compound Treatment

Human PCa cell lines PC3 and human normal hepatic cell line LO2, which were purchased from American Type Culture Collection, were stored in The Key Laboratory of Chemistry for Natural Products of Guizhou Province and Chinese Academic of Sciences (Guiyang, China). The PC3 cells were maintained in DMEM that was supplemented with 10% fetal bovine serum (FBS), 1% Penicillin-Streptomycin Solution (1 × 10^5^ U/L penicillin, 100 mg/L Streptomycin), and cultured at 37 °C in a CO_2_ incubator (5% CO_2_, 95% air, and 95% humidity). The DMEM was changed every one to two days. The cell growth state was observed under the microscope, and a cell passage could be carried out after growing them by 80–90% in the petri dish. After splitting them three times, the cells reached the logarithmic growth phase; the cells were used for subsequent experiments. The compound that was selected is an MTS derivative, named HXL131, with a purity of more than 99%. HXL131 was dissolved in DMSO to prepare a 2 × 10^4^ µmol/L stock solution.

### 4.4. Cell Growth Assay

#### 4.4.1. Cell Proliferation Assay

The effect of HXL131 on PC3 cell proliferation was determined by an MTT colorimetric assay which was conducted by referring to the given method in [58]. Briefly, PC3 cells at the logarithmic growth stage were plated into 96-well plates with a density of 5 × 10^3^ cells/well and placed in a cell incubator for overnight culture to make them adhere to the wall. The experimental group was used a gradient concentration of HXL131 (0.625, 1.25, 2.5, 5, 10 µmol/L), and 0.1% DMSO was used as the control group. Five samples were repeated in the wells which were set at each concentration. PC3 cells were treated for 24 h, 48 h, and 72 h in a cell incubator, and an inverted fluorescence microscope was used to observe the number and morphology of cells. Twenty µL MTT (5 mg/mL) was added to each well for 4 h, the supernatant was discarded by centrifugation, and 150 µL DMSO was added to a shaker for 15 min. A microplate reader detected the absorbance at 490 nm, and the inhibition rate of cell proliferation was calculated. Inhibition rate of the cell proliferation = 1 − (Average optical density of experimental group/Average optical density of control group) × 100%.

#### 4.4.2. Cell Apoptosis Assay

The effects of HXL131 on PC3 cell apoptosis were detected by using Annexin V-fluorescein isothiocyanate (FITC) and propidium iodide (PI) staining kits. The PC3 cells were inoculated into 6-well plates with 3 × 10^5^ cells/well and incubated with HXL131 (2.5, 5, 10 µmol/L) and 0.1% DMSO for 24 h. Cells were digested and collected with trypsin without ethylenediamine tetraacetic acid (EDTA), washed twice with phosphate-buffered saline (PBS), and mixed with 500 µL PBS to form cell suspension. Five µL Annexin V-FITC and 5 µL PI were added to avoid light staining for 30 min. The apoptosis rate of the cells in each concentration group was quantitatively detected by employing flow cytometry.

#### 4.4.3. Cell Cycle Assay

The effects of HXL131 on the PC3 cell cycle were detected by using an RNase A and PI staining kit. The procedure to inoculate and treat the cells is described in “Section 4.4.2”. The cells were digested and collected with trypsin-containing EDTA and fixed at 4 °C for more than 4 h with 70% precooled ethanol. They were washed with PBS twice, 5 µL RNase A was added to a 37 °C water bath for 30 min, then 25 µL PI solution was added at room temperature to avoid light staining for 15 min. The effect of different concentrations of HXL131 on cell cycle change was quantitatively detected by employing flow cytometry.

### 4.5. Cell Migration Assay

#### 4.5.1. Wound-Healing Assay

A wound-healing assay examined the effect of HXL131 on PC3 cell migration, and this was performed by us referring to the method that is described in [59]. Briefly, the cells were seeded at 1 × 10^6^ to 6-well plates using an FBS-free medium. When the confluence was greater than 90%, a sterile 200 µL pipette tip was used to create a wound in the PC3 cell monolayer, and detached cells were gently washed with PBS. Next, different concentrations of HXL131 were added. To avoid cell migration inhibition that is caused by high concentrations of HXL131, we used the HXL131 with a lower concentration gradient (1.25, 2.5, and 5 µmol/L). Images were taken at 0, 24, and 48 h after the scratch, and the wound-healing rates in each group were calculated using Image J software (V1.8.0.112, NIH, Bethesda, MD, USA).

#### 4.5.2. Trans-Well Chamber Migration Assay

The trans-well chamber detected the effect of HXL131 on PC3 cell migration. The cell density was adjusted to 2 × 10^5^ cells/mL in the DMEM without FBS. A two hundred µL cell suspension was added into the upper chamber of the trans-well chamber, and 600 μL DMEM containing 10% FBS was added into the lower chamber. After the cells had adhered to the wall, HXL131 (1.25, 2.5, 5 µmol/L) was added into the upper chamber and incubated for 24 h. The cells that did not migrate to the bottom of the trans-well chamber were wiped with cotton swabs, soaked in 70% methanol for 20 min to fix the migrated cells, stained with 0.1% crystal violet for 20 min, and washed with PBS and dried. The number of migrated cells was randomly observed under the microscope and photographed in three fields. The formula for the migration of the cells is as follows: inhibition rate of cell migration = 1 − (Average number of migrated cells in the experimental group/Average number of migrated cells in the control group) × 100%.

#### 4.5.3. Real-Time Quantitative Cell Migration Assay

The effects of HXL131 on PC3 cell migration were monitored using RTCA technology. Referring to the protocol in [60], PC3 cells were cultured in advance with FBS-free DMEM to induce the cell starvation effect. One hundred and sixty-five µL DMEM containing FBS was added to the lower chamber of the RTCA CIM-plate 16-well Plate to induce chemotaxis, and 30 µL DMEM free FBS was added to the upper chamber, and this placed in an incubator for 1 h to soak the membrane of the chamber. The cells were collected and inoculated into the upper chamber of the detection plate with 6 × 10^4^ cells/well and incubated at room temperature for 30 min. HXL131 (2.5, 5, 10, 15 µmol/L) was added as the experimental group, and 0.1% DMSO was used as the control group. The cell migration process of PC3 was monitored using the xCELLigence RTCA DP cell analyzer, and the real-time kinetic curve of cell migration was obtained.

### 4.6. Proteomic Bioinformatics Analysis of TMT Labeling

PC3 cells were treated with 5 µmol/L HXL131 (experimental group) and 0.1% DMSO (control group) for 24 h. The cell lysate was collected, and the proteins were extracted as described in the reference literature [61] and hydrolyzed into peptides by trypsin digestion. HPLC was used to classify the peptides, and LC-MS/MS was used to identify and quantify the proteome of the samples. Then, a Bioinformatics analysis was performed on the qualified proteins that were detected by mass spectrometry. A GO secondary annotation, subcellular localization, and COG/KOG were used to classify the functions of the DEPs. The function of the DEPs was analyzed from GO classification, KEGG pathways, and protein domain perspectives. A PPI network was constructed with the STRING database, and a visualization analysis was performed.

### 4.7. DEP Validation Assay

#### 4.7.1. GEPIA Assay

GEPIA (http://gepia.cancer-pku.cn/) in The Cancer Genome Atlas (TCGA) database was used to analyze the differential expression of the abovementioned DEPs in the PCa tissues and normal adjacent tissues. After collecting the data, a GraphPad Prism 7.0 was used for the statistical analysis.

#### 4.7.2. Western Blot Assay

Further, a Western blot was used to verify the results of the proteomics of the DEPs and CETSA analysis. The experiment was performed as previously described in [58]. Briefly, the cells were cultured in dishes and treated with different concentrations of HXL131 for 24 h. The cells were collected, an RIPA buffer containing 1% protease inhibitor PMSF was added, and the cells were lysed at 4 °C for 30 min. Then, the protein supernatant was collected, and the cell fragments were removed by centrifugation at 12,000 rpm for 20 min at 4 °C. The BCA protein determination kit quantitatively detected the protein concentration. The protein of each sample was determined to be 50 µg, and they were separated by 10% SDS-PAGE, and transferred to a 0.22 µM polyvinylidene fluoride (PVDF) membrane. The PVDF membrane was sealed in the buffer containing 5% non-fat milk in Tris-buffer saline, containing 0.1% Tween (TBST), and incubated at room temperature for 2 h. Next, the membrane was incubated overnight at 4 °C with the following primary antibodies: anti-CYR61, anti-TIMP1, anti-SOD2, anti-IL6, anti-SERPINE2, anti-DUSP1, anti-TNFSF9, anti-OSMR, anti-TNFRSF10D, anti-TNFRSF12A, and anti-GAPDH (loading control). The membranes were washed with TBST and incubated with goat anti-rabbit IgG H + L secondary antibodies. An Odyssey Infrared Imaging System detected the immunoreactive proteins, and the protein expression levels of these genes that were involved were measured by the gray-scale value of straps using Image J software.

#### 4.7.3. Molecular Docking Assay

Molecular docking was used to verify the targeted binding between the target proteins and small-molecule compound HXL131, and the stable binding protein was selected for a visual analysis. The 2D structure of HXL131 was drawn using the ChemBioDraw 14 software, and the 2D structure of HXL131 was converted to the 3D structure using the ChemBio3D Ultra software (V14.0.0.117, Cambridge, MA, USA), and the minimum free energy was realized. The UniProt database was used to find the entry number of the docking target. According to the entry number, the 3D structure of the critical target in PDB format was downloaded from the PDB database (https://www.rcsb.org/). The water molecules and small-molecule ligands were removed using the PyMOL software (V2.5, DeLano Scientific LLC). AutoDockTools software (V1.5.7, The Scripps Research Institute, San Diego, CA, USA) was used to add hydrogen ions and determine the active pocket position of the target protein. AutoDockVina software (V1.2.0, The Scripps Research Institute, CA, USA) was used for molecular docking, and the maximum energy difference was set as 5. Finally, PyMOL and Discovery Studio Client software (V4.5, Shenzhen, China) were used for visual processing of the results with solid binding force.

#### 4.7.4. CETSA Assay

An CETSA was used to verify the binding results of the compound and target protein in the molecular docking. PC3 cells were treated with HXL131 in a 10-fold gradient change (0.01, 0.1, 1, 10, 100 µmol/L, and the experimental group) and 0.1% DMSO (control group) for 2 h. The cells were collected and prepared into suspension with PBS. The suspension was placed in a metal bath at 53 °C for 3 min and then they were stood at room temperature for 2 min. The liquid nitrogen—37 °C water bath was cycled 3 times. Once it was centrifuged at 12,000 rpm for 15 min, the supernatant was discarded, and cell masses were collected. Follow-up steps are as described in Section 4.7.2.

#### 4.7.5. Gene Silencing Assay

The DUSP1 and TNFSF9 gene-silenced plasmids (Table 3) were constructed (Guizhou Hejin Biotechnology Co., Ltd., Guizhou, China) to validate the effect of silencing these two target genes on the growth and metastasis of the PC3 cells. The shDUSP1 1#, shDUSP1 2#, shDUSP1 3#, shTNFSF9 1#, shTNFSF9 2#, shTNFSF9 3#, and shVector plasmids were transfected into PC3 cells, and 48 h after transfection, a RT-qPCR (primer sequences are shown in Table 4) was performed to test whether these two genes were successfully silenced, the more successful one to silence the sequence was selected for subsequent validation experiments.

### 4.8. Statistical Analysis

IBM SPSS Statistics 21.0 (Chicago, IL, USA) and GraphPad Prism 7.0 (San Diego, CA, USA) statistical software was used for the data analysis. Data were presented as means ± SD of three independent experiments. Multiple *t*-tests, a one-way ANOVA, and a two-way ANOVA were used for the statistical analysis. *p* < 0.05 was considered statistically significant; n.s was considered non-significant.

## Figures and Tables

**Table 1:** Differentially expressed proteins between two samples under a fold change > 2.0 (*p*-value < 0.05).

Type of Regulation	Protein Symbol
Up	CYR61, IRAK2, B4GALT5, LYPD3, PLAU, TIMP1, FOS, HLA-A(68), MT-ND2, MT-ATP8, SOD2, IL6, JUN, HLA-A(24), SERPINE2, CXCL8, RALA, CEBPB, EFNA1, PTX3, DUSP1, EPHA2, TM4SF1, SLC7A1, SDC4, HRH1, TNFSF9, CLK1, STC1, OLR1, PLAUR, ZFP36L1, LAMB3, TNIP1, PCOLCE, PDK4, LY6K, DOLPP1, ITPRIP, HLA-B, SLC38A2, OSMR, B3GNT5, TMEM222, TNFRSF10D, TNFRSF12A, KLHL21
Down	TPM2, GNS, JPT1

**Table 2:** Molecular docking results of 10 key expressed proteins.

Target Symbol	Entry	PDB ID	Binding Affinity (kcal/mol)
CYR61	O00622	5WTT	−6.9
TIMP1	P01033	3V96	−7.8
SOD2	P04179	1PL4	−7.2
IL6	P05231	7NXZ	−6.0
SERPINE2	P07093	4DY0	−7.3
DUSP1	P28562	6D66	−8.7
TNFSF9	P41273	6MGP	−8.4
OSMR	Q99650	NA	NA
TNFRSF10D	Q9UBN6	NA	NA
TNFRSF12A	Q9NP84	2KMZ	−5.6

**Table 3:** The target site sequences were used in this study.

Target Name	Target Sequence
ShDUSP1 1#	GCTCTGTCAACGTGCGCTTCA
ShDUSP1 2#	CAAAGGAGGATACGAAGCGTT
ShDUSP1 3#	AGTTTGTGAAGCAGAGGCGAA
ShTNFSF9 1#	TGAGCTACAAAGAGGACACGA
ShTNFSF9 2#	CGAGGCTCGGAACTCGGCCTT
ShTNFSF9 3#	CCCTTCACCGAGGTCGGAATA
shVector	TTCTCCGAACGTGTCACGTAA

**Table 4:** The sequences of primers used in this study.

Gene Name	Primer Direction	Primer Sequence
DUSP1	Forward	5′-AGAGCCCCATTACGACCTCT-3′
	Reverse	5′-CCAGAGGAACTCGGGTGAAG-3′
TNFSF9	Forward	5′-AAATGTTCTGATCGATGGG-3′
	Reverse	5′-CCGCAGCTCTAGTTGAAAGAAGA-3′
GAPDH	Forward	5′-GGAGCGAGATCCCTCCAAAAT-3′
	Reverse	5′-GGCTGTTGTCATACTTCTCATGG-3′

## Data Availability

The data presented in this article are available.
